# Spatial Architecture of B7-H3-Expressing Cell Subpopulations Predicts Patient Prognosis in Lung Cancer Brain Metastases: A Pilot Study

**DOI:** 10.3390/cancers18142252

**Published:** 2026-07-14

**Authors:** Shigeaki Nawa, Mitsugu Fujita, Masasuke Ohno, Shunichiro Kuramitsu, Shota Nohira, Ryuta Saito

**Affiliations:** 1 Department of Neurosurgery, Nagoya University Graduate School of Medicine, 65 Tsurumai-cho, Showa-ku, Nagoya 466-8560, Aichi, Japan; snawa-spr@umin.ac.jp (S.N.); nohirashota.ncu@gmail.com (S.N.); saito.ryuta.b1@f.mail.nagoya-u.ac.jp (R.S.); 2 Department of Neurosurgery, Aichi Cancer Center, 1-1 Shikanodono, Chikusa-ku, Nagoya 464-8681, Aichi, Japan; 3 Center for Medical Education and Clinical Training, Kindai University Faculty of Medicine, 1-14-1 Miharadai, Minami-ku, Sakai 590-0197, Osaka, Japan; 4 Department of Neurosurgery, Nagoya Medical Center, 4-1-1 Sannomaru, Naka-ku, Nagoya 460-0001, Aichi, Japan; skramitz@yahoo.co.jp; 5 Department of Neurosurgery, Kariya Toyota General Hospital, 5-15 Sumiyoshi-cho, Kariya 448-8502, Aichi, Japan

**Keywords:** B7-H3, lung cancer brain metastasis, immune microenvironment, spatial analysis, prognosis

## Abstract

Background: The clinical outcomes of lung cancer brain metastases (LCBMs) are highly variable. Traditional pathology relies on bulk cell densities. These static measures fail to capture the spatial architecture of the tumor immune microenvironment (TIME). B7-H3 (CD276) represents a key immune checkpoint in LCBMs. We investigated whether the spatial orchestration of B7-H3-expressing cell populations is associated with patient prognosis. Methods: Multiplex immunohistochemistry (mIHC) for B7-H3 and Iba1 (a macrophage marker) was performed on surgically resected tissues from 22 patients, with single-cell segmentation and classification in QuPath. We then applied spatial point pattern and spatial autocorrelation analyses to evaluate the relative positioning of single cells, computing spatial interaction metrics, including cross-Moran’s I and the cross-K function, within a 35 μm radius. These metrics were correlated with postoperative overall survival (OS), and prognostic thresholds were determined via time-dependent ROC curve analysis. Results: Standard cell densities generally did not correlate with OS, although B7-H3^+^ tumor-associated macrophage (TAM) density showed a moderate positive correlation ($r_{s}=0.434$, *p* = 0.044), of a magnitude comparable to the clinical GPA score ($r_{s}=0.464$, *p* = 0.029). Conversely, specific spatial metrics emerged as candidate prognostic factors. High spatial mixing and clustering of B7-H3^+^ and B7-H3^−^ TAMs, as shown by high cross-Moran’s I (*p* = 0.011) and the cross-K function (*p* = 0.033), associated with significantly shorter OS. This pattern suggests a coordinated local immunosuppressive network. Conversely, high spatial integration between B7-H3^+^ and B7-H3^−^ tumor cells was associated with prolonged OS (*p* = 0.016), whereas spatial segregation of B7-H3^+^ tumor cells was associated with poor outcomes. Conclusions: Decoding the spatial architecture of B7-H3-expressing cell subpopulations provides superior prognostic stratification compared with standard density-based metrics. These localized spatial niches represent potential biomarkers and therapeutic targets for personalized LCBM management.

## Simple Summary

Brain metastases from lung cancer carry a poor, highly variable prognosis, yet routine pathology counts how many cells are present without considering where those cells sit relative to one another. In this exploratory pilot study of 22 patients, we asked whether the spatial arrangement of cells expressing the immune checkpoint B7-H3 carries prognostic information. Using multiplex staining and spatial statistics, we mapped B7-H3 on tumor cells and on tumor-associated macrophages (TAMs). Simple cell counts are rarely tracked with survival. By contrast, cell geometry are: when B7-H3-positive and B7-H3-negative TAMs were tightly intermixed, patients survived for a shorter time, pointing to a local immunosuppressive network; when positive and negative tumor cells were well intermixed, patients survived longer. These findings suggest that the spatial organization of B7-H3-expressing cells, rather than their abundance alone, may help refine prognosis in lung cancer brain metastases.

## 1. Introduction

Lung cancer remains the most frequently diagnosed malignancy and the leading cause of cancer-related death worldwide, accounting for approximately 2.5 million new cases and 1.8 million deaths annually, with incidence and mortality varying across regions according to tobacco exposure, ambient air pollution, and screening implementation [1]. Tobacco smoking remains the dominant risk factor, together with radon, occupational carcinogens, and particulate air pollution; nonetheless, a substantial proportion of cases, particularly among East Asian never-smokers, arise through oncogenic driver alterations such as epidermal growth factor receptor (EGFR) mutations [2]. Histologically, lung cancer comprises non-small-cell lung cancer (NSCLC; approximately 85% of cases) and small-cell lung cancer (SCLC; approximately 15%), which differ markedly in biology, treatment response, and prognosis [3]. Lung cancer is also the leading cause of brain metastases (BMs), which affect up to 40% of patients and cause severe neurological impairment and short overall survival (OS) [4,5].

The formation of lung cancer brain metastases (LCBMs) reflects a multistep metastatic cascade. Circulating tumor cells must intravasate, survive in the circulation, arrest within the cerebral microvasculature, breach the blood–brain barrier, and adapt to the neural niche to establish macrometastases [6]. Colonization depends on interactions with brain-resident astrocytes and microglia and on recruited bone marrow-derived macrophages, which together establish an immunosuppressive, myeloid-rich microenvironment distinct from the primary lung tumor [7]. These tissue-specific adaptations shape therapeutic response and prognosis, and warrant characterization of the local immune architecture of LCBMs.

Current prognostic stratification of LCBMs relies largely on clinical indices and bulk molecular markers. The Graded Prognostic Assessment (GPA) score [8] and its molecular update (Lung-molGPA), which incorporates programmed death-ligand 1 (PD-L1) expression and driver-mutation status [9], integrate systemic and clinical variables to estimate survival. Predictive biomarkers such as the PD-L1 tumor proportion score and actionable driver alterations (e.g., EGFR and anaplastic lymphoma kinase [ALK]) guide systemic therapy [10]. However, these tools depend on bulk characteristics or systemic variables and show limited reproducibility and prognostic resolution at the individual level [11]. Importantly, they disregard the spatial organization of the tumor immune microenvironment (TIME). Biomarkers of local cellular interaction could improve risk stratification.

Immune checkpoint molecules critically shape the TIME and constitute leading therapeutic targets. Beyond the programmed cell death 1 (PD-1)/PD-L1 axis that underpins current immunotherapy, tumors co-opt CTLA-4, LAG-3, TIM-3, and TIGIT to restrain anti-tumor immunity [12,13]. Among these, B7-H3 (CD276) is a type I transmembrane glycoprotein of the B7 immunoglobulin superfamily that is minimally expressed in normal tissues yet markedly overexpressed across solid tumors, conferring a favorable, tumor-selective therapeutic window [14]. Functionally, B7-H3 suppresses T-cell activation, promotes pro-tumorigenic macrophage phenotypes, and drives proliferation, invasion, and therapy resistance through both immunologic and non-immunologic mechanisms [15]. B7-H3 is expressed on both tumor cells and tumor-associated macrophages (TAMs), where it promotes tumor progression and immune evasion [16,17]. Although several studies have examined its prognostic impact in brain metastases, most have relied on bulk metrics such as overall positivity percentages [18], which overlook the substantial intratumoral heterogeneity and spatial organization of the brain microenvironment. These properties, namely tumor-selective expression, dual presence on tumor cells and TAMs, and active clinical-stage targeting, motivated our focus on B7-H3 rather than on more broadly expressed checkpoints.

Spatial biology has emerged as a powerful approach in biomedical research, demonstrating that the functional state of the TIME depends on cellular neighborhood interactions rather than on cell abundance alone [19,20]. Technologies for single-cell spatial mapping span antibody-based multiplex imaging, including multiplex immunohistochemistry (mIHC), imaging mass cytometry, and co-detection by indexing (CODEX), as well as spatial transcriptomic platforms, each resolving the position of individual cells within intact tissue [21]. Downstream, spatial point-pattern and spatial autocorrelation statistics quantify how phenotypes are arranged relative to one another, converting cell coordinates into interpretable interaction metrics. In cancer, such neighborhood analyses can add prognostic information beyond density-based measures [20]. The physical proximity between immunosuppressive myeloid cells and tumor cells governs anti-tumor immunity [22], yet within the myeloid-rich brain microenvironment the local cell-to-cell interactions involving B7-H3 remain poorly resolved, a gap that reflects broader challenges in adapting computational pathology to the brain [23].

To address this gap, we combined mIHC with spatial point-pattern and spatial autocorrelation statistics to map B7-H3-expressing subpopulations in surgically resected LCBMs. We hypothesized that the physical proximity of B7-H3^+^ and B7-H3^−^ subpopulations, rather than their absolute abundance, shapes the local immunosuppressive TIME and associates with OS. The specific aims of this exploratory pilot study were threefold: (i) to map the single-cell spatial distribution of B7-H3^+^ and B7-H3^−^ tumor cells and TAMs; (ii) to quantify their localized interactions using cross-Moran’s I, the cross-K function, and co-occurrence probability; and (iii) to test whether these spatial metrics associate with postoperative OS more informatively than conventional cell densities. By resolving cell-to-cell organization beyond the reach of static density analysis, we sought to generate spatially defined, hypothesis-generating prognostic candidates for LCBMs.

## 2. Materials and Methods

### 2.1. Patients

This single-center retrospective cohort study was approved by the Institutional Review Board of the Aichi Cancer Center (ACC; approval number: IR071501) in accordance with the Declaration of Helsinki. The requirement for individual informed consent was waived due to the retrospective design, and an opt-out procedure was provided on the institutional website in accordance with Japanese ethical guidelines. The inclusion criteria for the study were as follows: (i) age ≥ 20 years; (ii) histologically confirmed primary lung cancer; (iii) surgical resection of brain metastases performed at ACC; and (iv) available formalin-fixed paraffin-embedded (FFPE) tissue samples of sufficient quality for multiplex immunohistochemistry. The exclusion criteria were: (i) severe perioperative complications; or (ii) pathologically confirmed radiation necrosis without viable tumor cells.

During the study period from April 2018 to October 2024, a total of 27 consecutive patients underwent craniotomy and surgical resection for clinically suspected LCBMs at ACC. Upon central pathological review of the resected specimens, 5 patients (18.5%) were diagnosed with radiation necrosis without any viable tumor cells. In these 5 patients, who had received prior radiotherapy (stereotactic radiosurgery or whole-brain radiotherapy) for brain metastases, the radiation necrosis was preoperatively suspected as tumor recurrence or progression, prompting surgical resection. Because these 5 cases lacked viable tumor cells required for the spatial analysis of the tumor immune microenvironment, they were excluded from this study. Consequently, the remaining 22 patients with histologically confirmed viable LCBMs were included in the final analysis. The 22 included patients are characterized by age, sex, performance status, smoking history, histological subtype (non-small-cell lung cancer and small-cell lung cancer), mutational status, and prior treatment modalities. Histologically, the cohort comprised 20 patients with NSCLC, 1 with SCLC, and 1 with carcinoma, not otherwise specified (NOS). This cohort partially overlapped with our previous study on tertiary lymphoid structures [24] but remains strictly independent in target molecules (B7-H3/Iba1) and spatial statistical methodologies.

### 2.2. Multiplex Immunohistochemistry (mIHC)

We performed sequential chromogenic mIHC protocols using formalin-fixed, paraffin-embedded (FFPE) sections [25,26]. Deparaffinized and rehydrated 4-μm-thick sections underwent heat-induced epitope retrieval (HIER) in Tris-EDTA buffer (pH 9.0) at 121 °C for 20 min, followed by blocking with 3% hydrogen peroxide and a protein-blocking solution. We sequentially incubated sections with rabbit monoclonal antibodies against B7-H3 (clone EPR20115; Abcam, Cambridge, UK, 1:500) and Iba1 (clone EPR16589; Abcam, Cambridge, UK, 1:1000). We visualized signals using Histofine Simple Stain MAX-PO (peroxidase) and AP (alkaline phosphatase) systems (Nichirei Biosciences, Tokyo, Japan), developing chromogens sequentially with HistoGreen (B7-H3; Cosmo Bio, Tokyo, Japan) and First Red II (Iba1; Nichirei Biosciences, Tokyo, Japan); dark brown signal overlap identified B7-H3^+^ TAMs. Between rounds, we stripped antibodies using HIER in citrate buffer (pH 6.0) at 95 °C for 10 min, and verified stripping efficiency by omitting the primary antibody in subsequent runs to confirm the absence of residual signal. To ensure uniformity, we processed all sections simultaneously in a single batch via an automated stainer, followed by hematoxylin counterstaining.

### 2.3. Digital Pathology and Cell Detection

We performed image acquisition and analysis in accordance with previous protocols [24,27]. Following mIHC, we scanned whole-slide digital images via a NanoZoomer-SQ system (Hamamatsu Photonics, Hamamatsu, Japan) at 20× magnification (0.75 NA) and performed quantitative image analysis using QuPath software (version 0.7.0) [28]. We initially identified tumor regions using a threshold-based pixel classifier on the hematoxylin channel, manually refining these regions to exclude necrosis, hemorrhage, and artifacts (Supplementary Figure S1). For single-cell classification, we trained a supervised Random Trees classifier via a specimen-by-specimen active learning approach, progressively annotating phenotypes across all 22 specimens to generate a robust, unified model applied uniformly across the cohort. This classifier categorized single cells into four subpopulations: B7-H3^+^ tumor cells, B7-H3^−^ tumor cells, B7-H3^+^ TAMs, and B7-H3^−^ TAMs. Expert neuro-oncologists (S.N. and M.O.), blinded to clinical outcomes, visually verified the classification accuracy across all specimens based on morphological and phenotypic criteria. Final results were expressed as cell densities (cells/mm^2^ of tumor area).

### 2.4. Statistical Analysis of Spatial Architecture

We analyzed the TIME spatial architecture using the Python-based framework Squidpy (version 1.2.2) [29], which integrates SciPy (version 1.18.0rc1) and NetworkX (version 3.6.1) for spatial graph construction. We extracted the Cartesian coordinates of classified single cells to construct spatial maps and built spatial graphs to evaluate cellular interactions. To visualize phenotypic clustering, we performed Uniform Manifold Approximation and Projection (UMAP) based on morphological and multiplex marker intensity features (mean nuclear and cytoplasmic intensities for hematoxylin, B7-H3, and Iba1) obtained from cell segmentation.

Three complementary metrics captured distinct spatial dimensions: cross-Moran’s I for phenotypic spatial autocorrelation, the cross-K function for physical clustering, and co-occurrence probability for distance-dependent enrichment. We computed cross-Moran’s I and co-occurrence probability via Squidpy and calculated the cross-K function using the R package spatstat (version 3.6-1) [30]. First, we calculated cross-Moran’s I to assess B7-H3 phenotypic alignment among neighboring cells, utilizing an inverse-distance weight matrix ($1/d^{2}$) and a 199-iteration permutation test. Next, we calculated the cross-K function to quantify the physical aggregation of different cell types with Ripley’s isotropic edge correction. Because edge corrections and asymmetric cell densities can cause practical asymmetry, we designated the first cell type as the reference and the second as the target, reporting results in reference-to-target notation. Finally, we computed co-occurrence probability to measure the conditional likelihood of finding a target cell type at a given distance from a reference cell type relative to complete spatial randomness.

To correlate these spatial metrics with clinical outcomes, we selected a localized spatial radius of 35 μm as the standard representative threshold. This distance lies within the biologically plausible range for short-range paracrine signaling. Although a single secreting cell can in principle influence neighbors across a domain of up to approximately 250 μm under idealized diffusion [31], the effective spread of inflammatory cytokines such as interferon-gamma (IFN-γ) reaches only approximately 30–40 μm in tumor tissue [32], consistent with the 35 μm radius adopted here. Specifically, we extracted the values of all three metrics calculated at this 35 μm radius for downstream clinical statistical analysis. To ensure robustness against threshold bias, we performed sensitivity analyses across spatial radii of 10–50 μm in 5 μm increments.

### 2.5. Statistical Analysis of Clinical Data

We conducted clinical statistical analyses using EZR (Easy R) version 1.70 (Saitama Medical Center, Jichi Medical University, Japan) [33], a graphical user interface for R (version 4.5.0) [34], in accordance with statistical frameworks from previous studies [35,36,37]. Given the limited sample size (*N* = 22), we designed this study as an exploratory, hypothesis-generating analysis. OS was defined as the interval from the date of surgery to death from any cause; patients who remained alive at the data cutoff were censored at their last follow-up, and no patient was lost to follow-up. The median follow-up was 39.0 months, estimated using the reverse Kaplan–Meier method. To evaluate continuous associations between spatial metrics, clinical scores (such as the GPA score), and postoperative OS duration (months), we assessed normality using the Shapiro–Wilk test. Because the variables violated normality, we selected Spearman’s rank correlation as a non-parametric screen for monotonic associations and reported the rank correlation coefficients ($r_{s}$); we interpreted coefficients of approximately 0.4 as moderate associations. To establish optimal prognostic thresholds, we generated time-dependent receiver operating characteristic (ROC) curves at the median postoperative OS, evaluating both directions (higher values indicating higher risk, or vice versa) to maximize the area under the curve (AUC). The optimal cutoff was determined using the Youden index, and continuous variables were dichotomized into high and low groups. We then calculated hazard ratios (HRs) and 95% confidence intervals (CIs) via univariate Cox proportional hazards regression, compared survival curves using the Kaplan–Meier method with the log-rank test, and defined statistical significance as a *p*-value of <0.05. We did not perform multivariable Cox regression because the limited number of events relative to candidate covariates would not support a reliable adjusted model; consequently, all survival associations are unadjusted and exploratory. For the same reason, and because the three spatial metrics were pre-specified as biologically complementary readouts of a single neighborhood hypothesis, we did not apply formal correction for multiple comparisons. We therefore interpret all associations as hypothesis-generating and requiring validation in larger, independent cohorts, while noting that convergence across these independent metrics reduces the likelihood of spurious inference.

## 3. Results

### 3.1. Marked Spatial Heterogeneity of B7-H3-Expressing Populations Delineates the LCBM Microenvironment

**Figure 1:**
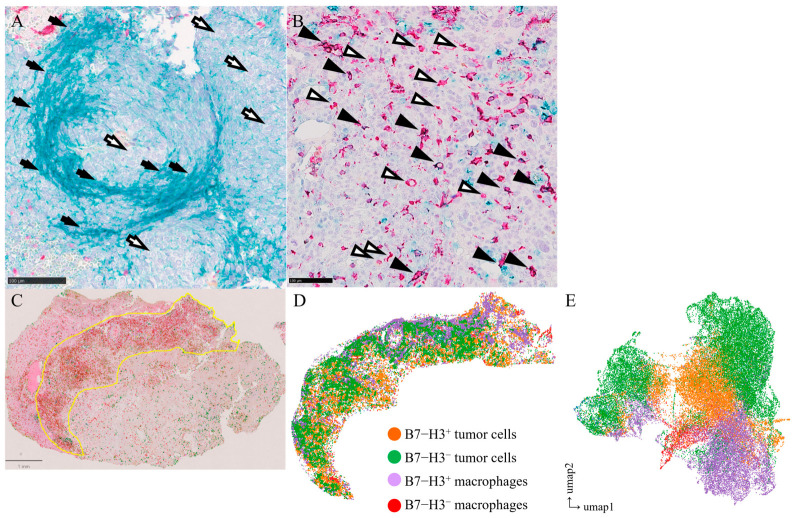
Representative multiplex immunostaining and spatial profiling images in lung cancer brain metastasis (LCBM). (**A**,**B**) Representative multiplex immunohistochemistry images of LCBM tissues stained for Iba1 (red) and B7-H3 (green) to visualize spatial heterogeneity. (**A**) Identification of B7-H3^+^ tumor cells (black arrows) and B7-H3^−^ tumor cells (white arrows). (**B**) Identification of B7-H3^+^ TAMs (black arrowheads) and B7-H3^−^ TAMs (white arrowheads); B7-H3^+^ TAMs appear dark brown owing to the spatial overlap of the red First Red II (Iba1) and green HistoGreen (B7-H3) chromogens (additional high-magnification examples in Supplementary Figure S2). Scale bar = 100 μm. (**C**) Representative digital pathology image demonstrating cell segmentation and classification using QuPath, with tumor regions defined by yellow regions of interest (ROI). (**D**) Spatial cell map visualizing the precise coordinates and distribution of the classified cell populations within the refined tumor region of interest (ROI) used for spatial analysis; the whole-section tissue context and the derivation of this ROI are shown in Supplementary Figure S1A. (**E**) Uniform Manifold Approximation and Projection (UMAP) plot displaying distinct cell clusters based on their multiplex marker expression profiles.

This study included 22 patients with LCBMs (Table 1). mIHC targeting B7-H3 and Iba1 revealed marked spatial heterogeneity within the TIME of LCBMs (Figure 1 and Supplementary Figure S2). Specifically, mIHC delineated distinct tumor parenchyma regions of B7-H3^+^ tumor cells (Figure 1A, black arrows) and B7-H3^−^ tumor cells (Figure 1A, white arrows). Furthermore, we distinguished infiltrating TAM populations into B7-H3^−^ (Figure 1B, white arrowheads) and B7-H3^+^ subpopulations (Figure 1B, black arrowheads) based on signal colocalization.

Following digital slide scanning and cell segmentation, Random Trees classifier performance stabilized after approximately 10 iterations, ensuring uniform cell classification. We extracted coordinates to construct spatial maps visualizing the single-cell topography (Figure 1D). Finally, UMAP analysis confirmed distinct phenotypic clustering of the identified cell populations based on multiplex marker profiles (Figure 1E).

### 3.2. Localized Spatial Interactions of Myeloid and Tumor Compartments Predict Overall Survival

**Figure 2:**
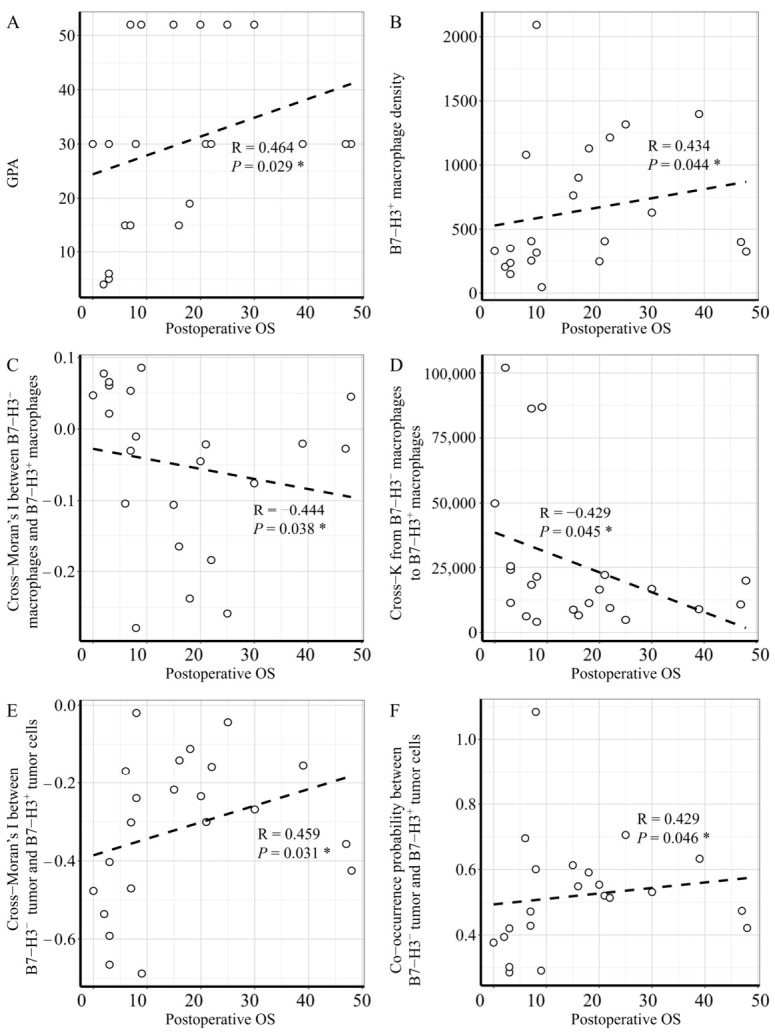
Scatter plots showing associations between clinical, pathological, and spatial metrics and postoperative overall survival (OS) in lung cancer brain metastasis (LCBM). Scatter plots illustrating Spearman’s rank correlations between postoperative OS in months and specific clinical, density-based, or spatial metrics evaluated within a 35 μm radius. The dashed lines indicate fitted lines. (**A**) Graded Prognostic Assessment (GPA) score. (**B**) Cell density of B7-H3^+^ TAMs (number of cells per mm^2^). (**C**) Cross-Moran’s I between B7-H3^−^ and B7-H3^+^ TAMs. (**D**) Cross-K function from B7-H3^−^ TAMs to B7-H3^+^ TAMs. (**E**) Cross-Moran’s I between B7-H3^−^ and B7-H3^+^ tumor cells. (**F**) Co-occurrence probability between B7-H3^−^ and B7-H3^+^ tumor cells. Each dot represents an individual patient (*N* = 22). Spearman’s rank correlation coefficient ($r_{s}$) and *p*-values are shown for significant associations (* *p* < 0.05).

This analysis evaluated the relationship between cellular metrics and postoperative OS (individual patient details in Table 2). Using a 35 μm spatial radius, we calculated Spearman’s rank correlation coefficients between spatial metrics and OS. As a clinical control, the GPA score showed a moderate, significant correlation with OS ($r_{s}=0.464$, *p* = 0.029; Figure 2A). While absolute cell densities generally did not correlate with OS (Supplementary Figure S3), B7-H3^+^ TAM density showed a moderate positive correlation ($r_{s}=0.434$, *p* = 0.044; Figure 2B).

Beyond abundance, several spatial interaction metrics showed moderate but significant associations with clinical outcomes, of a magnitude comparable to the clinical GPA score. Within the TAM compartment, proximal interactions negatively correlated with OS, including cross-Moran’s I between B7-H3^−^ and B7-H3^+^ TAMs (Figure 2C) and the cross-K function from B7-H3^−^ to B7-H3^+^ TAMs (Figure 2D). Conversely, in the tumor compartment, both cross-Moran’s I (Figure 2E) and co-occurrence probability (Figure 2F) between B7-H3^−^ and B7-H3^+^ tumor cells exhibited significant positive correlations with OS.

### 3.3. Spatial Neighborhood Profiling Offers Superior Prognostic Stratification over Cell Density

**Figure 3:**
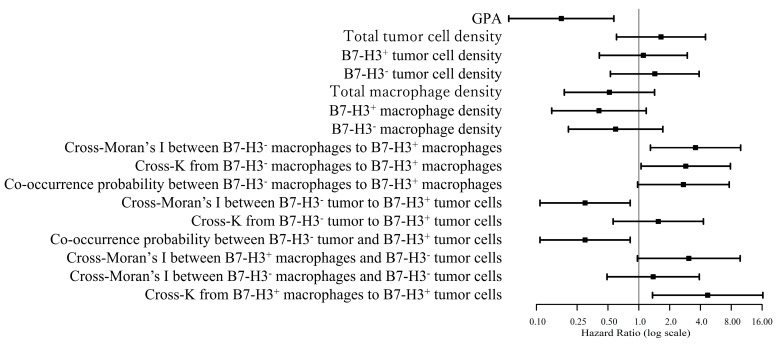
Univariate Cox proportional hazards analysis for postoperative overall survival (OS). Forest plot displaying the hazard ratios (HR) and 95% confidence intervals (CI) for cell density-based pathological metrics and spatial metrics (*N* = 22). Continuous variables were dichotomized into high and low groups based on the threshold values derived from the time-dependent receiver operating characteristic (ROC) curve analysis evaluated at the median postoperative OS. A vertical line at HR = 1 indicates no effect.

To evaluate the prognostic impact of these metrics, we performed a time-dependent ROC curve analysis at the median postoperative OS to determine optimal prognostic cutoff values (Table 3). Univariate Cox proportional hazards analysis indicated that, while simple cell densities did not associate with OS, specific spatial metrics emerged as candidate prognostic factors (Figure 3), particularly spatial metrics between B7-H3^+^ and B7-H3^−^ TAMs, and B7-H3^+^ and B7-H3^−^ tumor cells.

**Figure 4:**
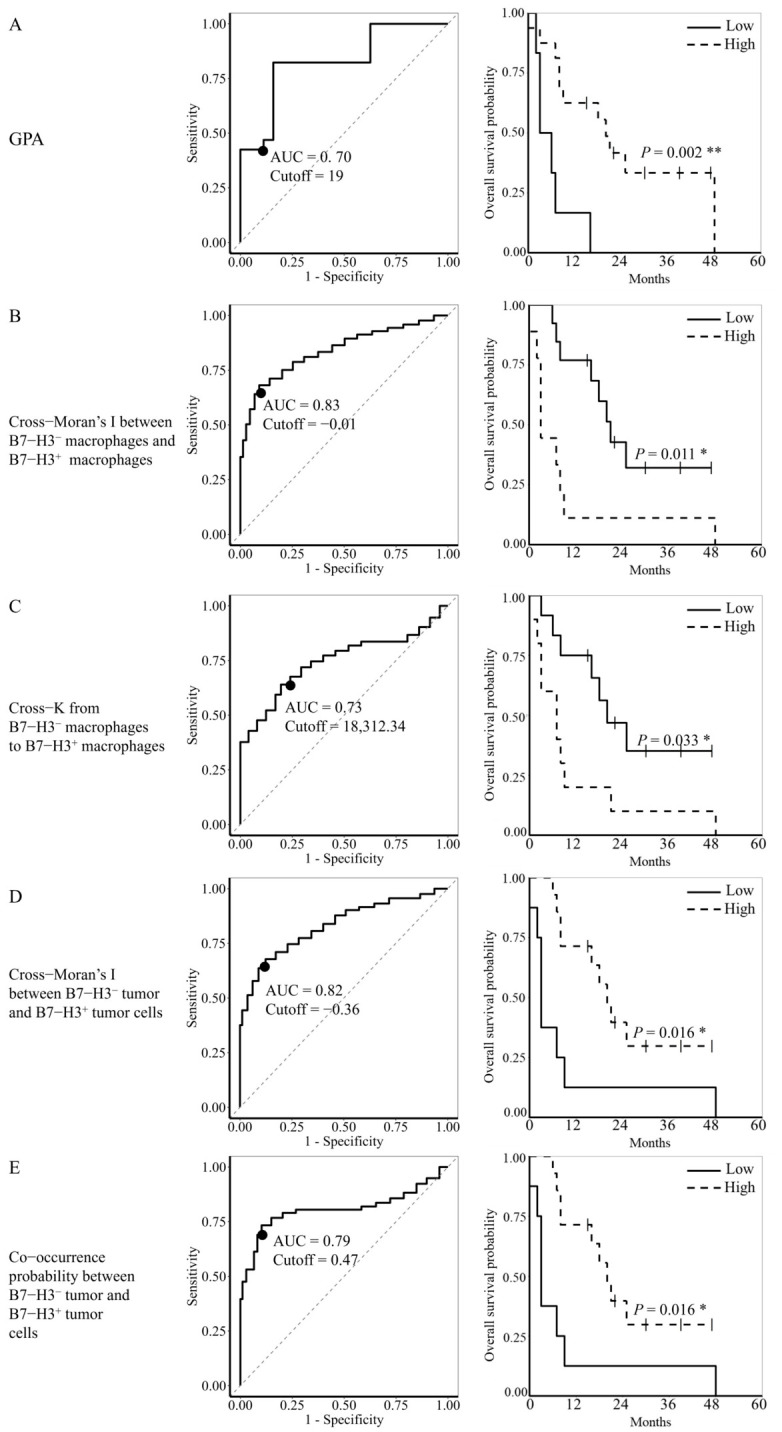
Prognostic stratification of postoperative overall survival (OS) using clinical and spatial metrics. Time-dependent receiver operating characteristic (ROC) curves (**left**) and corresponding Kaplan–Meier survival curves (**right**) illustrating the prognostic stratification of the cohort (*N* = 22). The optimal cutoff thresholds for each metric were established using ROC curve analysis evaluated at the median postoperative OS. The dashed diagonal lines represent the line of no discrimination (AUC = 0.5). Patient groups were subsequently dichotomized into ‘High’ and ‘Low’ group to assess differences in OS. The evaluated metrics include the clinical (**A**) Graded Prognostic Assessment (GPA) score, and spatial interaction parameters: (**B**) cross-Moran’s I between B7-H3^+^ and B7-H3^−^ TAMs, (**C**) cross-K function from B7-H3^−^ TAMs to B7-H3^+^ TAMs, (**D**) cross-Moran’s I between B7-H3^+^ and B7-H3^−^ tumor cells and (**E**) co-occurrence probability between B7-H3^+^ and B7-H3^−^ tumor cells. Statistical significance between stratified groups was determined using the log-rank test (* *p* < 0.05, ** *p* < 0.01).

Kaplan–Meier survival analysis stratified by the ROC-derived cutoffs further highlighted the prognostic relevance of spatial organization. The clinical GPA score achieved significant prognostic stratification (*p* = 0.002; Figure 4A). Patients with high spatial mixing and clustering of TAMs, indicated by high cross-Moran’s I (*p* = 0.011; Figure 4B) and cross-K function (*p* = 0.033; Figure 4C) between B7-H3^+^ and B7-H3^−^ TAMs, experienced significantly shorter OS. Conversely, higher cross-Moran’s I (*p* = 0.016; Figure 4D) and co-occurrence probability (*p* = 0.016; Figure 4E) between B7-H3^+^ and B7-H3^−^ tumor cells is associated with improved OS, which reflects non-random phenotypic mixing. In contrast, spatial segregation of B7-H3^+^ tumor cells are associated with poor outcomes.

## 4. Discussion

We characterized the TIME landscape in LCBMs by analyzing B7-H3 expression in tumor cells and TAMs with mIHC and spatial statistics (Figure 1). Spatial orchestration, rather than absolute density, is associated with postoperative OS (Figure 2 and Figure 3). Specifically, clustering and close proximity of B7-H3^+^ and B7-H3^−^ TAMs within 35 μm are associated with shortened OS (Figure 4B,C), suggesting a coordinated local immunosuppressive niche, whereas spatial segregation between B7-H3^+^ and B7-H3^−^ tumor cells is associated with poorer outcomes (Figure 4D,E). The significant correlation between the clinical GPA score and OS (Figure 2A) [8,9] serves as an important clinical control, confirming that our pilot cohort represents a standard LCBM population and supporting the translational relevance of these findings [38].

Our spatial analysis revealed marked segregation of B7-H3 expression into distinct positive and negative parenchymal regions (Figure 1A,E). Infiltrating TAMs likewise comprised distinct B7-H3^+^ and B7-H3^−^ subpopulations with clear spatial compartmentalization (Figure 1B). Although spatial transcriptomics has demonstrated macrophage accumulation and fibrogenic niches in LCBMs [39], it has not resolved the single-cell arrangement of B7-H3^+^ and B7-H3^−^ subpopulations. Our single-cell mapping addresses this gap, showing that B7-H3 expression forms localized microenvironmental domains with regionalized checkpoint interactions.

Spatial interactions played divergent prognostic roles across cell compartments. Although B7-H3^+^ TAM density positively correlated with OS (Figure 2B), their proximal interactions correlated negatively, including cross-Moran’s I between B7-H3^+^ and B7-H3^−^ TAMs (Figure 2C) and the cross-K function from B7-H3^−^ to B7-H3^+^ TAMs (Figure 2D). This paradox suggests that a higher total density of B7-H3^+^ TAMs may reflect active immune influx, whereas their localized mixing and clustering correlate with shorter OS. Checkpoint-expressing TAMs are not uniformly immunosuppressive; their clinical impact depends on microenvironmental context, as certain subpopulations exhibit immunostimulatory phenotypes in solid tumors [40], and checkpoint molecules such as B7-H3 and PD-L1 co-occur on TAMs in settings linked to favorable rather than adverse outcomes [41]. Their spatial organization, rather than abundance alone, therefore governs their clinical significance.

Spatial clustering of TAMs has been reported to coordinate immunosuppressive states and drive pro-tumorigenic M2-like polarization and therapy resistance in other malignancies [42,43]. Our findings suggest that when B7-H3^+^ and B7-H3^−^ TAMs aggregate in close proximity, they may construct a coordinated local immunosuppressive niche. Within such a neighborhood, B7-H3^+^ TAMs could suppress neighboring active TAMs and other immune cells via short-range cytokine gradients or direct cell-to-cell contact, thereby neutralizing anti-tumor TAM activity. This mechanistic interpretation remains a hypothesis derived from our spatial associations rather than a directly demonstrated causal pathway.

Conversely, within the tumor compartment, both cross-Moran’s I (Figure 2E) and co-occurrence probability (Figure 2F) between B7-H3^+^ and B7-H3^−^ tumor cells exhibited significant positive correlations with OS. These results contrast with conventional cell density assessments, which ignore spatial patterns and often yield conflicting prognostic value [44]. Across other malignancies, spatial parameters, such as T-cell clustering or tumor-TAM proximity, consistently outperform simple cell counts in predicting OS [45,46]. These cross-cancer observations support the superiority of spatial architecture over bulk density. Assessing the TIME through three complementary spatial metrics captured distinct dimensions of the microenvironment, and their alignment reinforces the validity of these observations: the cellular configurations represent physically and phenotypically coordinated units that are associated with OS.

Our univariate Cox analysis indicated an advantage of spatial metrics over conventional density-based measures (Figure 3), consistent with the limitations of bulk profiling [19,22]. Previous studies using simple density or percentage thresholds for B7-H3 have yielded inconsistent prognostic outcomes across solid tumors [16,17]. These discrepancies likely arise because static counts ignore tissue organization and intercellular communication that requires physical proximity. Cells must lie close together to interact via membrane-bound ligands or short-range paracrine gradients; for instance, the effective diffusion distance of inflammatory cytokines such as IFN-γ is confined to approximately 30–40 μm in tumor tissues [32]. Spatial architecture analysis resolves these microenvironmental interactions.

This spatial analysis offers insights into immune evasion within the brain microenvironment [47,48]. High spatial mixing of TAM subpopulations are associated with significantly shorter OS (Figure 4B,C). B7-H3 suppresses T-cell activation and promotes pro-tumorigenic TAM phenotypes [49,50]. Close proximity allows B7-H3^+^ TAMs to suppress adjacent B7-H3^−^ TAMs via paracrine factors (e.g., IL-10, TGF-β, CCL2) and neutralize anti-tumor TAM activity. TAM-derived IL-10 can also upregulate B7-H3 expression, forming a feedback loop that reinforces localized immune evasion [49] and shields tumor cells from cytotoxic T-cell infiltration [51].

Conversely, spatial patterns of tumor cells showed distinct prognostic implications: high spatial integration between B7-H3^+^ and B7-H3^−^ tumor cells is associated with prolonged OS (Figure 4D,E), whereas segregation is associated with poor outcomes. This segregation likely reflects localized clonal selection or adaptation to regional microenvironmental pressures that create therapy-resistant niches shielded from immune surveillance [52]. High spatial integration, by contrast, dilutes the local immunosuppressive barrier and allows effector immune cells to infiltrate and eliminate tumor cells.

Spatial metrics reached significance asymmetrically across compartments: physical clustering metrics (cross-K function) were highly discriminative in the TAM compartment (Figure 4C), whereas conditional proximity metrics (co-occurrence probability) were more informative in the tumor compartment (Figure 4E). This divergence may reflect the spatial constraints of each cell type: tumor cells formed dense, cohesive sheets occupying most of the parenchyma (Figure 1A), whereas infiltrating TAMs were sparsely dispersed or localized in small clusters (Figure 1B). For highly packed point patterns like tumor cells, physical clustering metrics such as Ripley’s K function can suffer from a ceiling or saturation effect that limits their capacity to resolve OS differences [19,30]. In this high-density compartment, the probabilistic mixing and phenotypic patterns of B7-H3^+^ and B7-H3^−^ subpopulations (reflected by co-occurrence probability and cross-Moran’s I) likely provide more sensitive indicators of local phenotypic heterogeneity (Figure 4D,E) [39]. For sparsely distributed immune populations, by contrast, physical aggregation itself represents a major functional shift that makes clustering metrics highly discriminative of localized immunosuppressive networks (Figure 4C) [22,42].

From a translational perspective, these findings suggest that spatial neighborhood profiling could serve as a valuable tool for precision oncology. B7-H3 represents a promising therapeutic target, with several monoclonal antibodies (e.g., enoblituzumab), antibody-drug conjugates (ADCs) (e.g., ifinatamab deruxtecan [DS-7300]), and chimeric antigen receptor T (CAR-T) cell therapies in active clinical development [53,54]. Because standard stratification based on bulk B7-H3 expression may overlook the spatial organization of target cells, patients with segregated, highly clustered B7-H3^+^ tumor niches or coordinated B7-H3^+^ and B7-H3^−^ TAM networks might exhibit distinct therapeutic susceptibilities. The spatial geometry of B7-H3-expressing populations could therefore serve as a predictive companion biomarker for B7-H3-targeted therapies and combination immunotherapies. Realizing this at scale will likely require brain-optimized computational pathology that integrates lineage-specific myeloid phenotyping with topological TIME analysis, an approach proposed conceptually for metastatic brain tumors [23]. To our knowledge, this study is the first to resolve the single-cell spatial architecture of B7-H3^+^ and B7-H3^−^ subpopulations in LCBMs and to link their localized organization to postoperative OS, positioning spatial neighborhood profiling as a conceptual advance over bulk B7-H3 quantification.

Our study has several limitations, and we therefore frame it as an exploratory, hypothesis-generating pilot study. First, the small sample size (N=22) and few events precluded multivariable Cox regression to adjust for clinical confounders, including the intensive multimodal therapy (immune checkpoint inhibitors, targeted agents, and radiation or surgery) the cohort received. Accordingly, the reported associations are unadjusted, and we cannot determine whether spatial architecture predicts survival independently of treatment regimens or other prognostic factors; the significant GPA correlation indicates that these spatial metrics provide complementary, rather than established independent, prognostic information. Second, we applied no formal correction for multiple comparisons, so the associations require confirmation in larger cohorts. Third, the cohort combined histologic subtypes that differ in biology and prognosis; it was predominantly NSCLC (20 of 22 patients), with a single SCLC case and a single carcinoma, not otherwise specified (NOS) case. In an exploratory re-analysis restricted to the 20 NSCLC patients (Supplementary Figure S4), the univariate Cox associations were metric-specific: those based on cross-Moran’s I and co-occurrence probability remained significant, whereas those based on the cross-K function lost significance in three of the four previously significant comparisons, with one reversal in the hazard ratio direction (B7-H3^+^ macrophages to B7-H3^+^ tumor cells: HR 4.71 in the full cohort versus 0.36 in the NSCLC-only subgroup). The threshold-dependent analyses (ROC-derived cutoffs, Spearman correlation, and log-rank tests) shifted accordingly. This instability was specific to the cross-K estimates, which are sensitive to edge effects and local point density at this sample size, rather than a uniform subtype effect; the study was not powered for definitive subtype-stratified inference. Fourth, the retrospective, single-center design may introduce selection bias; the cohort comprised Japanese surgical candidates for LCBM resection, most with a smoking history (59% former smokers), so the findings may not generalize to unresectable, non-surgical, or non-Japanese populations. Fifth, we did not calculate formal classifier validation metrics, such as sensitivity and specificity, on an independent cohort, although expert visual verification supported objective cell counting. In addition, TAMs were defined by Iba1 as a pan-macrophage marker with B7-H3, without functional or polarization markers (such as CD163, CD206, or CD68); the immunosuppressive character attributed to B7-H3^+^ TAM neighborhoods is therefore inferred from the immunobiology of B7-H3 and its spatial associations with OS, rather than directly demonstrated, and requires confirmation with dedicated functional multiplex panels. Moreover, Iba1 does not distinguish resident microglia from bone marrow-derived macrophages, so our TAM compartment may conflate these functionally opposing lineages, a limitation intrinsic to pan-macrophage markers in intracranial tissue [23]. Finally, we could not directly quantify local cytokine gradients or single-cell functional states; further high-dimensional spatial transcriptomic, proteomic, or mechanistic studies must validate these localized interactions [20].

## 5. Conclusions

Overall, this exploratory pilot study indicates that the spatial architecture of B7-H3-expressing cells captures prognostic features of the LCBM immune microenvironment that density-based metrics overlook. Combining multiplex immunohistochemistry with three complementary spatial statistics resolved two divergent patterns. In the myeloid compartment, close mixing and clustering of B7-H3^+^ and B7-H3^−^ TAMs are associated with shorter OS, consistent with a coordinated local immunosuppressive niche. In the tumor compartment, spatial integration of B7-H3^+^ and B7-H3^−^ tumor cells is associated with longer OS, whereas segregation of B7-H3^+^ tumor cells is associated with poorer outcomes. These spatial patterns, benchmarked against the GPA score as a clinical anchor, represent candidate biomarkers for prognostic stratification rather than established independent predictors. Because the analyses were unadjusted, uncorrected for multiple comparisons, and derived from a small single-center cohort, the findings are hypothesis-generating and require validation in larger, prospective, multi-institutional cohorts with subtype-stratified and multivariable analyses. If validated, spatial neighborhood profiling of B7-H3-expressing cells could complement existing clinical indices and guide personalized therapeutic strategies, including B7-H3-targeted therapies, for patients with LCBMs.

## Figures and Tables

**Table 1:** Clinical characteristics of patients with lung cancer brain metastases. Summary of baseline clinical demographics, pathological diagnosis, mutational status, and treatment modalities for the 22 patients included in the study cohort. ALK, anaplastic lymphoma kinase; BM, brain metastasis; ECM, extracranial metastasis; EGFR, epidermal growth factor receptor; GPA, Graded Prognostic Assessment; ICI, immune checkpoint inhibitor; KPS, Karnofsky Performance Status; KRAS, Kirsten rat sarcoma viral oncogene homolog; LCBM, lung cancer brain metastasis; MST, median survival time; NOS, not otherwise specified; OS, overall survival; PD-L1, programmed death-ligand 1; SRS, stereotactic radiosurgery; SRT, stereotactic radiotherapy; TPS, tumor proportion score; WBRT, whole-brain radiotherapy.

Patient Characteristics	Values
No. of patients	22
Age, years, median (range)	70 (41–83)
Sex, No. (%)	
Female	10 (45)
Male	12 (55)
Preoperative KPS, No. (%)	
90–100	8 (36)
70–80	9 (41)
≤60	5 (23)
KPS at BM diagnosis, No. (%)	
90–100	13 (59)
70–80	7 (32)
≤60	2 (9)
Number of BM at diagnosis, No. (%)	
≥5	3 (14)
1–4	19 (86)
0	0 (0)
ECM at BM diagnosis, No. (%)	
No	17 (77)
Yes	5 (23)
Pathological diagnosis, No. (%)	
Adenocarcinoma	19 (86)
Squamous cell carcinoma	1 (5)
Small cell carcinoma	1 (5)
Other epithelial carcinoma, NOS	1 (5)
Gene mutation status, No. (%)	
None	10 (45)
EGFR sensitizing mutation	6 (27)
EGFR exon 20 insertion	1 (5)
ALK rearrangement/fusion	2 (9)
KRAS	3 (14)
Preoperative corticosteroids, No. (%)	
No	7 (32)
Yes	15 (68)
Preoperative chemotherapy, No. (%)	
No	7 (32)
Yes	15 (68)
Postoperative chemotherapy, No. (%)	
No	8 (36)
Yes	9 (41)
Undetermined	4 (18)
Unknown	1 (5)
Targeted therapy, No. (%)	
No	10 (45)
Yes	9 (41)
Undetermined	3 (14)
PD-L1 TPS category, No. (%)	
0%	6 (27)
1–49%	12 (55)
Unknown	4 (18)
Preoperative ICI, No. (%)	
No	14 (64)
Yes	8 (36)
Preoperative radiation therapy, No. (%)	
None	8 (36)
SRS	5 (23)
SRT	8 (36)
WBRT	1 (5)
Postoperative radiation therapy, No. (%)	
None	7 (32)
SRS	1 (5)
SRT	5 (23)
WBRT	5 (23)
Unknown	4 (18)
Smoking status, No. (%)	
None	9 (41)
Former	13 (59)
Current	0 (0)
Smoking exposure, pack-years, No. (%)	
0	9 (41)
<20	4 (18)
≧20	9 (41)
GPA-predicted MST, months, median (range)	30 (4–52)
Postoperative OS, months, median (range)	12 (0–48)

**Table 2:** Pathological and spatial characteristics of the LCBM cohorts. Summary of absolute cell densities and calculated spatial metrics for each patient (*N* = 22). Densities are provided for B7-H3^+^ and B7-H3^−^ tumor cells and TAMs. Spatial metrics, including cross-Moran’s I, the cross-K function, and co-occurrence probability between specific cell subpopulations, were calculated using a defined spatial radius of 35 μm.

Sample No	Age (years)	Pathological Diagnosis	GPA	Total Tumor Density	B7-H3^+^ Tumor Cell Density	B7-H3^−^ Tumor Cell Density	Total Macrophage Density	B7-H3^+^ Macrophage Density	B7-H3^−^ Macrophage Density	Cross-Moran’s I Between B7-H3^−^ Tumor and B7-H3^+^ Tumor Cells	Co-Occurrence Probability Between B7-H3^−^ and B7-H3^+^ Tumor Cells	Cross-K from B7-H3^−^ to B7-H3^+^ Tumor Cells	Cross-Moran’s I Between B7-H3^−^ Macrophages to B7-H3^+^ Macrophages	Cross-K from B7-H3^−^ Macrophages to B7-H3^+^ Macrophages	Co-Occurrence Probability Between B7-H3^−^ Macrophages to B7-H3^+^ Macrophages	Cross-Moran’s I Between B7-H3^+^ Macrophages and B7-H3^−^ Tumor Cells	Cross-Moran’s I Between B7-H3^−^ Macrophages And B7-H3^−^ Tumor Cells	Cross-K from B7-H3^+^ Macrophages to B7-H3^+^ Tumor Cells
1	73	Adenocarcinoma	30	7596.18	2276.47	5319.71	932.23	322.3	609.93	−0.43	0.42	4582.94	0.05	19,904.6	2.71	−0.26	−0.38	1819.49
2	65	Adenocarcinoma	52	1736.65	429.06	1307.59	3763.14	1316.51	2446.63	−0.04	0.71	4347.21	−0.26	4835.19	0.63	−0.17	−0.4	3314.31
3	75	Adenocarcinoma	30	2156.42	906.98	1249.44	817.23	406.96	410.27	−0.3	0.52	8791.54	−0.02	22,089.5	1.26	−0.36	−0.32	6144.24
4	76	Adenocarcinoma	30	3340.69	1227.18	2113.51	905.61	314.1	591.51	−0.24	0.6	8497.39	−0.01	21,413.6	1.23	−0.28	−0.56	5777.81
5	68	Adenocarcinoma	52	2300.68	932.62	1368.06	525.19	246.27	278.92	−0.23	0.55	4774.19	−0.05	16,476.9	0.99	−0.3	−0.63	5081.5
6	77	Squamous cell carcinoma	5	7398.12	5695.11	1703.01	494.87	147.49	347.38	−0.67	0.29	2725.52	0.06	24,075.1	3.38	−0.11	−0.2	1652.84
7	77	Small cell carcinoma	4	10,185.43	4109.91	6075.52	983.53	203.56	779.98	−0.54	0.39	15,470.6	0.08	102,023	3.65	−0.15	−0.33	9891.62
8	75	Adenocarcinoma	30	3409.41	2403.41	1006	4230.49	2091.68	2138.81	−0.02	1.08	5102.39	−0.28	4082.25	0.74	−0.16	−0.24	1447.52
9	69	Adenocarcinoma	52	2686.88	1412.35	1274.53	883.01	407.44	475.57	−0.3	0.43	6223	−0.03	18,312.3	1.03	−0.29	−0.51	4366.15
10	73	Adenocarcinoma	15	3239.11	1131.66	2107.45	1764.14	1080.1	684.03	−0.17	0.7	4306.19	−0.1	6199.58	0.9	−0.37	−0.35	2542.73
11	70	Adenocarcinoma	6	5300.81	2092.08	3208.73	1161.91	348.97	812.95	−0.4	0.42	2358.89	0.02	11,379.8	1.63	−0.26	−0.57	1136.22
12	53	Adenocarcinoma	30	5499.58	3305.76	2193.82	492.25	233.8	258.45	−0.59	0.3	2397.98	0.07	25,469.4	1.95	−0.21	−0.38	3492.27
13	51	Adenocarcinoma	15	7047.23	2937.67	4109.56	601.02	251.86	349.17	−0.47	0.47	18,616.7	0.05	86,292.5	3.15	−0.23	−0.31	9234.88
14	41	Adenocarcinoma	30	4274.04	2326.06	1947.98	1238.18	400.91	837.27	−0.36	0.47	4003.7	−0.03	10,760.5	1.13	−0.2	−0.36	2669.77
15	56	Adenocarcinoma	52	6570.38	5233.94	1336.44	274.6	45.59	229.01	−0.69	0.29	6116.73	0.09	86,863.3	3.46	−0.07	−0.13	10,513.6
16	42	Carcinoma, NOS	19	2589.2	1133.93	1455.27	2829.83	1129.34	1700.49	−0.11	0.59	7396.92	−0.24	11,276.6	0.69	−0.21	−0.37	5360.6
17	61	Adenocarcinoma	30	5044.12	1092.28	3951.84	1976.42	1397.52	578.9	−0.15	0.63	3922.12	−0.02	8928.87	1.27	−0.48	−0.39	3201.88
18	69	Adenocarcinoma	52	2696.84	1466.74	1230.1	1048.7	630.13	418.57	−0.27	0.53	6404.21	−0.08	16,720.8	1.05	−0.36	−0.35	4005.15
19	76	Adenocarcinoma	30	5055.19	1976.48	3078.71	620.93	327.44	293.49	−0.48	0.38	10,222.6	0.05	49,871.8	2.52	−0.34	−0.35	7438.79
20	71	Adenocarcinoma	30	4167.77	3132.57	1035.2	2085.54	1215.06	870.49	−0.16	0.51	3967.8	−0.18	9383.82	0.76	−0.2	−0.23	3417.5
21	83	Adenocarcinoma	15	4141.63	3454.81	686.82	1830.91	901.67	929.25	−0.14	0.55	2463.4	−0.17	6590.11	0.81	−0.15	−0.25	1814.04
22	50	Adenocarcinoma	52	4293.31	2056.13	2237.18	1812.7	764.11	1048.59	−0.22	0.61	5027.9	−0.11	8765.7	0.85	−0.28	−0.38	3502.86

**Table 3:** Time-dependent receiver operating characteristic (ROC) curve analysis of clinical, cell density, and spatial metrics for postoperative overall survival (OS). Determination of prognostic cutoff values using time-dependent ROC curve analysis evaluated at the median postoperative OS (*N* = 22). The table presents the Area Under the Curve (AUC), optimal cutoff threshold, sensitivity, specificity, and Youden Index for the Graded Prognostic Assessment (GPA) score, simple cell density metrics, and spatial interaction metrics. Corresponding Hazard Ratios (HR) with 95% Confidence Intervals (CI) and *p*-values derived from univariate Cox proportional hazards analysis are also shown (* *p* < 0.05, ** *p* < 0.01). AUC, area under the curve; CI, confidence interval; GPA, graded prognostic assessment; HR, hazard ratio; ROC, receiver operating characteristic.

Markers	AUC	Cutoff	Sensitivity	Specificity	Youden Index	Hazard Ratio (95% CI)	*p*-Value
GPA	0.7	19	0.42	0.89	0.31	0.18 (0.05–0.57)	0.004 **
Total tumor cell density	0.71	4293	0.6	0.75	0.35	1.65 (0.61–4.47)	0.329
B7-H3^+^ tumor cell density	0.71	2056	0.64	0.64	0.28	1.11 (0.41–2.98)	0.84
B7-H3^−^ tumor cell density	0.67	1455	0.69	0.6	0.29	1.43 (0.53–3.88)	0.48
Total macrophage density	0.81	1161	0.79	0.7	0.5	0.52 (0.19–1.43)	0.202
B7-H3^+^ macrophage density	0.8	630	0.85	0.66	0.5	0.41 (0.14–1.18)	0.099 *
B7-H3^−^ macrophage density	0.72	812	0.8	0.52	0.32	0.59 (0.21–1.72)	0.336
Cross^−^Moran’s I between B7-H3^−^ macrophages to B7-H3^+^ macrophages	0.83	−0.01	0.64	0.91	0.55	3.58 (1.30–9.87)	0.014 *
Cross^−^K from B7-H3^−^ macrophages to B7-H3^+^ macrophages	0.73	18,312	0.64	0.76	0.4	2.87 (1.05–7.85)	0.039 *
Co^−^occurrence probability between B7-H3^−^ macrophages to B7-H3^+^ macrophages	0.77	1.95	0.5	0.95	0.45	2.72 (0.97–7.63)	0.057
Cross^−^Moran’s I between B7-H3^−^ tumor to B7-H3^+^ tumor cells	0.82	−0.36	0.64	0.88	0.51	0.30 (0.11–0.82)	0.02 *
Cross^−^K from B7-H3^−^ tumor to B7-H3^+^ tumor cells	0.61	6404	0.4	0.83	0.24	1.55 (0.56–4.27)	0.398
Co^−^occurrence probability between B7-H3^−^ tumor and B7-H3^+^ tumor cells	0.79	0.47	0.69	0.9	0.59	0.30 (0.11–0.82)	0.02 *
Cross^−^Moran’s I between B7-H3^+^ macrophages and B7-H3^−^ tumor cells	0.59	−0.16	0.27	0.91	0.18	3.08 (0.97–9.78)	0.056
Cross^−^Moran’s I between B7-H3^−^ macrophages and B7-H3^−^ tumor cells	0.6	−0.32	0.39	0.82	0.2	1.38 (0.49–3.90)	0.542

## Data Availability

The datasets generated and analyzed during the current study are not publicly available because of institutional policy and the risk of compromising patient privacy in this small cohort. De-identified data may be made available from the corresponding author upon reasonable request and with permission from the Institutional Review Board of ACC.

## Supplementary Materials

The following supporting information can be downloaded at: https://www.mdpi.com/article/10.3390/cancers18142252/s1, Figure S1: Workflow of tumor-region definition and manual refinement in QuPath, showing (A) initial identification of tumor regions and single cells, (B) the manually refined tumor region with necrosis, hemorrhage, and artifacts excluded, and (C) a high-magnification view of the boxed region confirming the refined boundary; Figure S2: Additional representative high-magnification multiplex immunohistochemistry (mIHC) fields (A–F) from multiple LCBM specimens stained for B7-H3 and Iba1; Figure S3: Correlations between cell densities and postoperative OS in LCBM; Figure S4: Univariate Cox proportional hazards forest plot for the NSCLC-only subgroup (n = 20).

## Abbreviations

The following abbreviations are used in this manuscript:

ACC	Aichi Cancer Center
ADC	Antibody-drug conjugate
AP	Alkaline phosphatase
AUC	Area under the curve
B7-H3	B7 homolog 3 (CD276)
BM	Brain metastasis
CAR-T	Chimeric antigen receptor T cell
CI	Confidence interval
CODEX	Co-detection by indexing
ECM	Extracranial metastasis
EDTA	Ethylenediaminetetraacetic acid
EGFR	Epidermal growth factor receptor
EZR	Easy R
FFPE	Formalin-fixed, paraffin-embedded
GPA	Graded Prognostic Assessment
HIER	Heat-induced epitope retrieval
HR	Hazard ratio
IFN-γ	Interferon-gamma
Iba1	Ionized calcium-binding adapter molecule 1
ICI	Immune checkpoint inhibitor
IRB	Institutional Review Board
KPS	Karnofsky Performance Status
LCBM	Lung cancer brain metastasis
mIHC	Multiplex immunohistochemistry
NOS	Not otherwise specified
OS	Overall survival
PD-1	Programmed cell death 1
PD-L1	Programmed death-ligand 1
PO	Peroxidase
ROC	Receiver operating characteristic
ROI	Region of interest
SRS	Stereotactic radiosurgery
SRT	Stereotactic radiotherapy
TAM	Tumor-associated macrophage
TIME	Tumor immune microenvironment
TPS	Tumor proportion score
UMAP	Uniform Manifold Approximation and Projection
WBRT	Whole-brain radiotherapy
